# Generation and characterization of cerebellar granule neurons specific knockout mice of Golli-MBP

**DOI:** 10.1007/s11248-024-00382-0

**Published:** 2024-04-29

**Authors:** Haruko Miyazaki, Saki Nishioka, Tomoyuki Yamanaka, Manabu Abe, Yukio Imamura, Tomohiro Miyasaka, Nobuto Kakuda, Toshitaka Oohashi, Tomomi Shimogori, Kazuhiro Yamakawa, Masahito Ikawa, Nobuyuki Nukina

**Affiliations:** 1 Laboratory of Structural Neuropathology, Graduate School of Brain Science, Doshisha University, 1-3 Tatara Miyakodani, Kyotanabe-shi, Kyoto, 610-0394 Japan; 2 Department of Molecular Biology and Biochemistry, Dentistry and Pharmaceutical Sciences, Okayama University Graduate School of Medicine, 2-5-1 Shikata-cho, Kita-ku, Okayama, 700-8558 Japan; 3 Department of Experimental Genome Research, Research Institute for Microbial Diseases, Osaka University, 3-1 Yamadaoka, Suita, Osaka 565-0871 Japan; 4 Department of Animal Model Development, Brain Research Institute, Niigata University, 1-757 Asahimachidori, Chuo-ku, Niigata, 951-8585 Japan; 5 Faculty of Life and Medical Sciences, Doshisha University, 1-3 Tatara Miyakodani, Kyotanabe-shi, Kyoto 610-0394 Japan; 6 Laboratory for Molecular Mechanisms of Brain Development, RIKEN Center for Brain Science, 2-1 Hirosawa, Wako-shi, Saitama, 351-0198 Japan; 7 Laboratory for Neurogenetics, RIKEN Center for Brain Science, 2-1 Hirosawa, Wako-shi, Saitama, 351-0198 Japan; 8 Department of Neurodevelopmental Disorder Genetics, Institute of Brain Sciences, Nagoya City University Graduate School of Medical Sciences, Nagoya, Aichi 467-8601 Japan; 9 Department of Neuroscience of Disease, Brain Research Institute, Niigata University, 1-757 Asahimachidori, Chuo-ku, Niigata, 951-8585 Japan

**Keywords:** Golli-MBP, Cerebellar granule neuron, CRISPR/Cas9, Conditional knockout

## Abstract

Golli–myelin basic proteins, encoded by the myelin basic protein gene, are widely expressed in neurons and oligodendrocytes in the central nervous system. Further, prior research has shown that Golli–myelin basic protein is necessary for myelination and neuronal maturation during central nervous system development. In this study, we established Golli–myelin basic protein-floxed mice to elucidate the cell-type-specific effects of Golli–myelin basic protein knockout through the generation of conditional knockout mice (*Golli*–*myelin basic proteins*^*fl/fl*^*; E3CreN*), in which Golli–myelin basic proteins were specifically deleted in cerebellar granule neurons, where Golli–myelin basic proteins are expressed abundantly in wild-type mice. To investigate the role of Golli–myelin basic proteins in cerebellar granule neurons, we further performed histopathological analyses of these mice, with results indicating no morphological changes or degeneration of the major cellular components of the cerebellum. Furthermore, behavioral analysis showed that *Golli*–*myelin basic proteins*^*fl/fl*^*; E3CreN* mice were healthy and did not display any abnormal behavior. These results suggest that the loss of Golli–myelin basic proteins in cerebellar granule neurons does not lead to cerebellar perturbations or behavioral abnormalities. This mouse model could therefore be employed to analyze the effect of Golli–myelin basic protein deletion in specific cell types of the central nervous system, such as other neuronal cells and oligodendrocytes, or in lymphocytes of the immune system.

**Supplementary Information:**

The online version contains supplementary material available at 10.1007/s11248-024-00382-0.

## Introduction

The myelin basic protein (MBP) gene encodes two families of proteins: Classic-MBP and Golli-MBP. Classic-MBP isoforms are the major myelin proteins in the central nervous system (CNS), and their expression is restricted to mature oligodendrocytes and myelin sheaths (Nave and Werner 2014). The *Shiverer (shi)* mouse, in which *the Mbp* gene is mutated, exhibits an absence of compact myelin (Readhead and Hood 1990), suggesting that Classic-MBP plays an essential role in myelin formation and compaction in the CNS.

Transcription of Golli-MBP messenger ribonucleic acid (mRNAs) begins upstream of the transcription start site of Classic-MBP mRNA, and alternative splicing yields Golli-MBP transcripts containing exons 1–3 (Harauz and Boggs 2013). Golli-MBP is widely expressed in the mature oligodendrocytes and neurons of adult mouse CNS (Campagnoni et al. 1993; Landry et al. 1996; Givogri et al. 2001), although Golli was originally designated as “**g**ene expressed in the **ol**igodendrocyte **li**neage.” During postnatal development in the mouse brain, Golli-MBP is widely distributed in axonal fibers, such as mossy fibers of the hippocampus, striatonigral projection fibers, and parallel fibers of the cerebellum (Landry et al. 1996). In the embryonic mouse brain, Golli-MBP is expressed in the axonal process and cell bodies of the neuronal population (Landry et al. 1998), as well as in immature oligodendrocytes, such as oligodendrocyte progenitor cells (Paez et al. 2009). These findings suggest that Golli-MBP is expressed in both oligodendrocytes and neurons in the mouse brain during development and adulthood.

To assess the role of Golli-MBP, Jacobs et al*.* generated a Golli-MBP knockout mouse in which Golli-MBP was selectively ablated without affecting Classic-MBP isoforms (Jacobs et al. 2005). These mice exhibited impaired myelin-like sheet formation in cultured oligodendrocytes, region-specific hypomyelination in the visual cortex, and altered calcium ions (Ca^2+^) homeostasis in cultured oligodendrocytes (Jacobs et al. 2005). These results suggest that Golli-MBP may play a role in myelination by regulating intracellular Ca^2+^ levels in oligodendrocytes. Further studies using Golli-MBP-knockout mice revealed that Golli-MBP functions as a modulator of voltage-operated Ca^2+^ channels (VOCCs) in neurons. Decreased intracellular Ca^2+^ influx was observed after plasma membrane depolarization in the cortical and hippocampal regions of Golli-MBP knockout mice, and this Ca^2+^ influx was abolished by treatment with a VOCC antagonist (Cheli et al. 2016). The overexpression of Golli-MBP proteins in cultured neurons enhances Ca^2+^ influx and promotes neurite outgrowth, suggesting that Golli-MBP affects neuronal outgrowth (Cheli et al. 2016). These results suggest that Golli-MBP modulates neuronal VOCCs.

Golli-MBP is abundantly expressed in both the immune and nervous systems. Golli-MBP proteins are found ubiquitously in the immune system, including in T lymphocytes, B lymphocytes, and macrophages (Feng 2007; Feng et al. 2004; Voskuhl 1998). Previous studies have demonstrated that Golli-MBP overexpression inhibits T-cell activation in Jurkat T-cells (Feng et al. 2004), and Golli-MBP-deficient T-cells have shown enhanced Ca^2+^ entry following T-cell receptor stimulation (Feng et al. 2006). These findings suggest that Golli-MBP negatively regulates T-cell activation by modulating Ca^2+^ influx, which is crucial for T-cell activation (Joseph et al. 2014).

In this study, we established Golli-MBP-floxed (*Golli-MBP*^*fl/fl*^) mice using the CRISPR/Cas9 system to investigate the cell type–specific functions of Golli-MBP. We generated *Golli-MBP*^*fl/fl*^*; E3CreN* mice, in which Golli-MBP was specifically ablated in cerebellar granule neurons (CGNs), where abundant Golli-MBP expression has been reported in the cell body and axon fibers (Landry et al. 1996). Immunohistochemical analysis revealed no obvious morphological changes in the cerebellum of *Golli-MBP*^*fl/fl*^*; E3CreN* mice, and behavioral analysis did not show any abnormal behavior, suggesting that the loss of Golli-MBP does not induce significant morphological and functional perturbations in the cerebellum.

## Materials and methods

### Animal experiments

All surgical procedures and experiments received approval from the Ethics Committee of Doshisha University (Number: A15083, for animal experiments; number: D15083, for recombinant genetic experiments). All animal experiments were performed in compliance with guidelines established by the Animal Experiments Committee of Doshisha University. The experiments were conducted in accordance with approved ethical guidelines and regulations.

### Construction of the targeting gene of Golli-MBP-floxed mouse

Golli-MBP-floxed mice were generated using CRISPR/Cas9 genome editing technology. Single-guide RNAs (sgRNAs) and PAM sites were designed using the CRISPRdirect web tool (http://crispr.dbcls.jp/) (Naito et al. 2015). CRISPR/Cas9 plasmids were prepared by ligating oligonucleotides for sgRNAs (Table S1) into the *Bbs*I site of pSpCas9(BB)-2A-Puro (PX459) V2.0 (Addgene number: 62988, Teddington, UK) (Ran et al. 2013). To select highly efficient sgRNAs, we prepared four sgRNAs (#1–4) for the left target locus (Target-left) and four sgRNAs (#5–8) for the right target locus (Target-right) (Fig. S1a). The sgRNA sequence was verified by sequencing with the pX459-seq primer (Table S1). Approximately 400 bp region of target locus was amplified by PCR using primers anchored with restriction sites of *EcoR*I (5’end) and *BamH*I (3’end) (Fig. S1a, Table S1). The amplified DNA fragments were digested using *EcoR*I and *BamH*I, purified using gel electrophoresis, and ligated into the *EcoR*I and *BamH*I sites of pCAG-EGxxFP (Addgene number: 50716). The resulting plasmid sequence was verified by sequencing using the pCAG EGxxFP-seq primer (Table S1). The cleavage efficiency of sgRNAs was evaluated by co-transfecting HEK293T cells with CRISPR/Cas9 plasmids containing sgRNAs and pCAG-EGxxFP plasmids containing the target sites of the sgRNAs, as previously described (Mashiko et al. 2013). Finally, sgRNA4 and sgRNA8 were selected as highly efficient sgRNAs based on EGFP fluorescence intensity (Fig. S1b). Images of the transfected HK293T cells were obtained using an EVOS Floid Cell Imaging Station (Thermo Fisher Scientific, Waltham, MA, USA). LoxP–MBP exon2–LoxP DNA composed of three fragments (left, center, and right) was amplified by PCR using the primers listed in Table S2. The left DNA fragment was amplified by PCR using primer Golli-Pr1 and Golli-Pr2 anchored with restriction sites of *Xba*I (5’ end) and *BamH*I (3’ end), respectively. The Center DNA fragment was amplified by PCR using loxP sequence containing primer Golli-Pr3 and Golli-Pr4 anchored restriction sites *BamH*I (5’ end) and *EcoR*I (3’ end), respectively. The right DNA fragment was amplified by PCR using primer Golli-Pr5 and Golli-Pr6 anchored restriction sites *EcoR*I (5’ end) and *Xho*I (3’ end), respectively (Fig. S2 and Table S2). The amplified left DNA fragment was digested using *Xba*I and *BamH*I, the central DNA fragment was digested using *BamH*I and *EcoR*I, and the right DNA fragment was digested using *EcoR*I and *Xho*I. DNA fragments were purified via gel electrophoresis, ligated into multiple cloning sites of pBlueScript SKII, and digested using compatible restriction enzymes (Fig. S2). The resulting plasmid sequence was verified by sequencing using the T7 and T3 primers (Table S2). CRISPR/Cas9/sgRNA (#4 or #8)–expressing plasmids and LoxP–MBP exon2–LoxP containing plasmid were co-transfected into a mouse ES cell line derived from C57BL/6N (EGR-101). Transfected ES cells were transiently selected with puromycin, and 96 resultant ES cell clones were verified initially via nested-PCR using specific primers (first PCR: Golli-PrF4 and Golli-PrR5, second PCR: Golli-PrF and Golli-PrR), and the amplified DNA fragments were digested using *BamH*I and *EcoR*I (Fig. S3 and Table S3). Five candidate ES clones (#8, # 31, # 38, # 40, and # 92) were confirmed to be correctly targeted by sequencing using the Golli-seq FW and Golli-seq RV primers (Fig. S3 and Table S3). After karyotype analysis, three clones, #8, # 38, and # 40, were selected for microinjection into 8-cell stage embryos of ICR mice to create Golli-MBP-floxed mice. Chimeric male mice, identified by dark pigmentation of the eyes and coat, were paired with C57BL6/J female mice for germline transmission. The genotype of the offspring was verified by PCR using primers Golli-PrF5 and Golli-PrR, and the amplified DNA fragments were digested using *EcoR*I and *BamH*I (Fig. S3 and Table S3). Germline transmission occurred in line #40 Golli-MBP-floxed mice. To confirm the sequence of 3’ and 5’ loxP sites in the floxed allele of the mouse, the tail DNA of line #40 Golli-MBP-floxed mouse was extracted. DNA fragments of 3' and 5' loxP sites were amplified by PCR using the following primer set (3' loxP site: Golli-Pr1 and Golli-Pr4; 5’ loxP site: Golli-Pr3 and Golli-Pr6) (Table S2). The sequences of the DNA fragments were verified by sequencing using Golli-seq FW and RV primers (Table S3). Golli-MBP-floxed mice were deposited at the RIKEN BioResource Research Center (Number: RBRC10380) and Kumamoto University Animal Facilities (Number: 2730).

Golli-MBP-floxed mice were identified via PCR using two specific primer sets: Golli-flox-FW1 and -RV1, Golli-flox-FW2 and -RV2 (Table S4) detect left and right loxP insertion individually. Genotyping PCR was conducted using TaKaRa Taq HS (#R007A, Takara, Shiga, Japan) and the following cycling parameters: pre-incubation (94 °C for 1 min), amplification-step (40 cycles of 94 °C for 30 s, 60 °C for 30 s and 72 °C for 30 s) and post-incubation (72 °C for 2 min). *Golli-MBP*^*fl/fl*^*; E3CreN* mice and their controls were generated by breeding *Golli-MBP*^*fl/fl*^ male mice with *Golli-MBP*^*fl/*+^*; E3CreN* female mice.

### Other animals

*Scn4b-Venus* transgenic mice, expressing Venus under the control of the 9-kb *Scn4b* promoter, were identified via PCR using specific primers (Table S4) that detected Venus (599 bp). Genotyping PCR was carried out using TaKaRa Taq HS and the following cycling parameters: pre-incubation (94 °C for 1 min), amplification (35 cycles of 94 °C for 30 s, 60 °C for 30 s and 72 °C for 1 min), and post-incubation (72 °C for 7 min).

*E3CreN* mice (Miyazaki et al. 2012), in which the Cre gene is expressed in CGNs under the control of the *Grin2c* promoter, were identified by PCR using specific primers (Table S4) that detected the wild-type allele (487 bp) and the iCre knock-in allele (189 bp). Genotyping PCR was carried out using KOD-Plus-Neo (#KOD-401, TOYOBO, Osaka, Japan) and following cycling parameters: pre-incubation (98 °C for 1 min), amplification (3 cycles of 98 °C for 15 s, 64 °C for 2 s and 74 °C for 3 min) followed by (5 cycles of 98 °C for 15 s, 60 °C for 2 s and 74 °C for 3 min), and (27 cycles of 98 °C for 15 s, 56 °C for 2 s and 74 °C for 3 min).

*Scn2a*-floxed mice (Ogiwara et al. 2018) were used to assess the Cre recombinase activity in *E3CreN* mice. Because Nav1.2 encoded by *Scn2a* gene is abundantly distributed in parallel fibers, which are the axons of CGNs, Cre recombinase activity was evaluated using Nav1.2 expression levels in the parallel fibers of *Scn2a*^*fl/fl*^*; E3CreN* mouse. *Scn2a*-floxed mice were identified by PCR using specific primers (Table S4) that detected the wild-type (925 bp), floxed allele (1,162 bp), and deleted alleles (284 bp). Genotyping PCR was performed using TaKaRa Taq HS and the following cycling parameters: pre-incubation (94 °C for 1 min), amplification-step (35 cycles of 94 °C for 30 s, 60 °C for 30 s and 72 °C for 1 min), and post-incubation (72 °C for 7 min). To generate *Scn2a*^*fl/fl*^*; E3CreN* mice, *Scn2a*^*fl/fl*^ male mice were bred with *Golli-MBP*^*fl/*+^*; E3CreN* female mice.

Ai14 Td tomato reporter mice were obtained from Jackson Laboratories (Stock No.: 007914). Ai14 was identified via PCR using specific primers (Table S4) that detected the wild-type (297 bp) and mutant alleles (200 bp). Genotyping PCR was conducted using TaKaRa Taq HS and the following cycling parameters: pre-incubation (94 °C for 2 min), amplification-step1 (10 cycles of 94 °C for 20 s, 65 °C for 15 s (− 0.5 °C/cycle) and 68 °C for 10 s), amplification-step2 (20 cycles of 94 °C for 15 s, 60 °C for 15 s and 72 °C for 10 s) and post-incubation (72 °C for 2 min). Ai14 homozygous mice were bred with E3CreN mice to produce double-heterozygous mice (*Ai14; E3CreN* mice).

Animals were acclimated to a 12-h light/dark cycle and had access to food and water ad libitum.

### Generation of anti-Nav1.2 antibody

A rat monoclonal anti-Nav1.2 (clone 1B6-1C1) was developed KLH-using conjugated oligopeptides that correspond to the human Nav1.2 amino acids 466–485 (ASAESRDFSGAGGIGVFSE) with an additional C at its N-terminus. Female rats (WKY/Izm) were immunized, and hybridoma production was conducted by the Cell Engineering Corporation (Osaka, Japan). Hybridoma supernatants were screened using ELISA with antigen peptides, resulting in 48 positive clones. Clone 1B6-1C1 was selected through further screening involving Western blotting and immunohistochemistry (IH), employing total mouse brain lysates and mouse brain sections, respectively. The conditioned media from the stable hybridoma culture were collected, and the antibody was purified using a HiTrap SP HP column (#17-1151-01, GE Healthcare, Chicago, USA).

### Antibodies

In this study, we utilized the following primary antibodies: anti-NeuN (mouse, 1:200, #MAB377, Chemicon); anti-DARPP-32 (rabbit, 1:300, #AB10518, Millipore, Burlington, MA, USA); anti-TPPP (rabbit, 1:2,000, #ab92305, Abcam, Cambridge, MA, USA); anti-Olig2 (rabbit, 1:500, #AB9610, Millipore); anti-Nav1.2 (clone 1B6-1C1) (rat, 1:700); anti-calbindin D28K (rabbit, 1:300, #AB1778, Chemicon); anti-VGlut1 (guinea pig, 1:200, #Vglut1-GP-Af570, FRONTIER INSTITUTE, Japan); anti-PSD95 (rabbit, 1:100, #51–6900, Thermo Fisher Scientifics); anti-PSD95 (rabbit, 1:1,000, #PSD95-Rb-Af1720, FRONTIER INSTITUTE); anti-MBP (mouse, 1:3,000, #SMI-94, COVANCE); anti-GFAP (rabbit, 1:1,000, #Nr. Z0334, DAKO); rabbit anti-IbaI (rabbit 1:1,000, #019–19741, Wako, Osaka, Japan). Additionally, we employed biotinylated anti-rat/mouse/rabbit/guinea pig IgG secondary antibodies at a 1:300 dilution (Vector Laboratories, Newark, CA, USA).

### Tissue preparation for histological analysis

Prior to surgical procedures, mice were anesthetized with a combination of medetomidine, midazolam, and butorphanol. The mice were then perfused with PBS, followed by 4% paraformaldehyde (PFA) in PBS. Mouse brains were subsequently dissected and postfixed overnight with 4% PFA in PBS. To prepare paraffin sections, the post-fixed mouse brains were dehydrated with ethanol, transferred to xylene, and embedded in paraffin. Sections, 4 µm in thickness, were obtained using the HM430 sliding microtome (Thermo Fisher Scientific). For frozen sections, mouse brains were transferred and immersed in 30% sucrose (w/v) in PBS and then embedded in OCT compound by immediate freezing with liquid nitrogen. The frozen brain samples were stored at − 80 °C, and either 20 µm- or 12 µm-thick frozen sections were cut using the Leica CM1850 Cryostat (Leica Biosystems, Wetzlar, Germany).

### In situ hybridization (ISH)

The cDNA fragments used as ISH probes for Classic-MBP and Golli-MBP were amplified using KOD-Plus-Neo polymerase with specific primers (Table S5). The amplified cDNAs were subcloned into the pcDNA™ 3.1/V5-His C vector (#V81020, Thermo Fisher Scientific), and their integrity was verified through sequencing. Digoxigenin (DIG)-labeled probes were synthesized using a MEGAscript™ T7 Transcription Kit (#AM1333, ThermoFisher Scientific) and DIG RNA Labeling Mix (#11277073910, Roche, Basel, Switzerland) according to the manufacturer’s instructions. DIG-labeled cRNA probes hybridized to 20 µm-thick frozen mouse brain sections, and signals were detected as previously described (Miyazaki et al. 2014).

### ViewRNA ISH Tissue 1-Plex Assay

The ViewRNA ISH Tissue 1-Plex Assay is a valuable method for visualizing target mRNA localization in tissue sections using highly specific and branched DNA signal amplification technology (ViewRNA Tissue 1 Plex Assay Kit: #TFA-QVT0050, ViewRNA Tissue 1 plex Signal Amplification Kit: TFA-QVT0200, Thermo Fisher Scientific). Golli-MBP specific TYPE1 (Red signal) probe sets based on the sequence within 869–4,820 bases of NM_001025245.1 (Mus musculus MBP, transcript variant 8, mRNA) were produced by VERITAS Co., Ltd (Tokyo, Japan). Frozen brain sections, 12 µm in thickness, were obtained from 8-week-old C57BL6/J mice. These sections underwent pretreatment with a protease solution at 40 °C for 40 min, followed by hybridized with Golli-MBP specific probe sets at 40 °C for 3 h. Subsequently, signals were detected and amplified using the Fast Red substrate in accordance with the manufacturer’s instructions. Following detection, the sections were counterstained with Mayer’s hematoxylin (#30002, Muto Pure Chemicals Co., Ltd., Tokyo, Japan) and mounted with UltraMount Permanent Mounting Medium (#S1964, DAKO) to fix Fast Red signals.

### IH

Paraffin-embedded mouse sections were deparaffined, rehydrated, and then autoclaved for 5 min at 120 °C utilizing pH 6.0 citrate buffer for antigen retrieval. For DAB (3, 3 –diaminobenzidine) staining, sections were incubated with 0.3% hydrogen peroxide for 30 min at room temperature to block endogenous peroxidase activity. Sections were incubated with a blocking solution containing 5% skim milk in TBST (20 mM Tris–HCl [pH 7.5], 150 mM NaCl, 0.05% Tween 20) for 1 h at room temperature, with primary antibodies in TBST overnight at 4 °C, followed by biotin-conjugated secondary antibodies for 3 h at room temperature. The sections were incubated with VECTASTAIN *Elite* ABC Reagent, Peroxidase, R.T.U. (#PK-7100, Vector Laboratories) for 30 min at room temperature, following which DAB solution was applied. After sufficient color development, the slides were washed, dehydrated, and mounted using a xylene-based mounting solution. The closed sections were counterstained with Carrazzi’s hematoxylin (#30022; Muto Pure Chemicals Co., Ltd.). For ISH-IH combined staining, after ISH detected the mRNA signals of Classic MBP and Golli-MBP, the sections were stained with anti-NeuN, anti-TPPP, or anti-Olig2. Antigen retrieval and blocking of endogenous peroxidase activity were not conducted. The sections were incubated with a blocking solution containing 5% skim milk in TBST, followed by incubation with primary and secondary antibodies. Signals were detected using DAB solution and mounted using the xylene-based mounting solution described above. Images were acquired using BZ-X710 (KEYENCE, Osaka, Japan).

### qPCR

Medium spiny neurons (MSNs) from 4-week-old *Scn4b-Venus* mice (N = 6, male) were obtained by FACS, as previously described (Miyazaki et al. 2020). Approximately 4–20 ng of total RNAs was extracted from the MSNs using an RNeasy Micro Kit (#74004, Qiagen, Hilden, Germany) following the manufacturer's instructions. cDNA was synthesized and amplified using the Ovation Pico WTA System V2 (#3302-12, NuGEN, San Carlos, CA, USA) per the manufacturer’s instructions. An input amount of 2 ng of total RNA was used for cDNA synthesis and amplification. We utilized 2 ng of cDNAs as the template for qPCR.

Approximately 20 µg total RNAs were extracted from the striatum of a 4-week-old wild-type mouse (N = 4, male), cerebellum of 8-week-old *Golli-MBP*^*fl/fl*^*; E3CreN, Golli-MBP*^*fl/*+^*; E3CreN* and its control (*Golli-MBP*^*fl/fl*^*; WT* and *Golli-MBP*^*fl/*+^*; WT*) (N = 5 from each genotype, male and female) using RNeasy Mini Kit (#74104, Qiagen) according to the manufacturer’s instructions. cDNAs obtained from total RNAs were synthesized using ReverTra Ace -alpha-® (#FSK-101, TOYOBO) following the manufacturer’s instructions (input amount of total RNA was 1 mg). Template cDNAs prepared at a 50-fold dilution were used for qPCR. Template cDNAs and specific primers were mixed with FastStart Universal SYBR Green Master (Rox) (#4913914001, Roche), and PCRs were performed by a LightCycler®480 Real-Time PCR System (Roche). Cycling parameters for PCR were as follows: pre-incubation (95 °C for 10 min), amplification (60 cycles of 95 °C for 15 s and 60 °C for 1 min), melting curve (65 °C–95 °C with a 0.5 °C increase in temperature every 5 s), and cooling (40 °C for 30 s). When comparing mRNA expression levels among the control, *Golli-MBP*^*fl/fl*^*; E3CreN* and *Golli-MBP*^*fl/*+^*; E3CreN*, standards were generated using the cDNA diluent of cerebellar samples. Target gene expression was normalized to that of *Gapdh* in each sample. The primers used for qPCR are listed in Table S5.

### Image analysis

Sagittal brain sections from 8- and 55-week-old *Golli-MBP*^*fl/fl*^*; E3CreN* mice and the corresponding controls (*Golli-MBP*^*fl/*+^*; WT* or *Golli-MBP*^*fl/fl*^*; WT*) were stained with hematoxylin and cell type-specific markers (anti-NeuN, anti-calbindin D28K, anti-Vglut1, anti-PSD95, and anti-IbaI antibody) for image analysis.

To analyze the thickness of the molecular layer (ML) and granule cell layer (GCL) in the cerebellum, three hematoxylin-stained images per mouse were captured on a BZ-X710 with a 20 × objective lens. We used the open-source software package ImageJ (Fiji) to measure the layer thickness. Moreover, the average values from three images were used for statistical analysis. To analyze the number of CGNs, three NeuN-stained IH images per mouse were captured using a BZ-X710 with a 20 × objective lens. NeuN-positive neurons in the 100µm x 100µm region of interest (ROI) in the GCL were counted using Fiji. The average values from three images were used for statistical analysis. To analyze the Purkinje cell number and cell body size, three calbindin D28K-stained IH images of per mouse were captured on a BZ-X710 with a 20 × objective lens. The number and cell body size of Purkinje cells in a 250 µm-layer length were counted and measured using Fiji. The number of Purkinje cells within the 250 µm-layer length was calculated to be within the 100 µm-layer length. The average value from three images per mouse was used for statistical analysis. To analyze the number of Vglut1-positive and PSD95-positive puncta, three Vglut1 and PSD95-stained IH images per mouse were captured on a BZ-X710 with a 60 × objective lens. To facilitate better visualization of puncta, we used Haze Reduction, a BZ-X710 analyzer software, with the following settings: blur size, 8; brightness, 7.0; and reduction rate, 0.9. Vglut1-positive and PSD95-positive puncta within the 50µm x 50µm ROI in the ML were counted using Hybrid Cell Count Application, a BZ-X710 analyzer software. The number of Vglut1- and PSD95-positive puncta within each ROI was calculated within 1µm^2^. The average value from three images per mouse was used for the statistical analysis. To analyze the number of IbaI-positive microglia, three IbaI-stained IH images per mouse were captured using a BZ-X710 with a 20 × objective lens. The number of IbaI-positive microglia within the ROI and selected areas of the ML or GCL in each image were counted using a Hybrid Cell Count Application. The number of IbaI-positive microglia within each ROI was calculated to be within 1mm^2^. The average value from three images per mouse was used for the statistical analysis.

### Body weight

We measured the body weight of older *Golli-MBP*^*fl/fl*^*; E3CreN, Golli-MBP*^*fl/*+^*; E3CreN,* and control (*Golli-MBP*^*fl/fl*^*; WT* and *Golli-MBP*^*fl/*+^*; WT*) mice aged 52–53 weeks because the adult *Golli-MBP*^*fl/fl*^*; E3CreN* mice (approximately 12-weeks-old) looked healthy and no apparent changes were observed compared with control mice.

### Rotarod test

Motor coordination and balance of *Golli-MBP*^*fl/fl*^*; E3CreN, Golli-MBP*^*fl/*+^*; E3CreN,* and control (*Golli-MBP*^*fl/fl*^*; WT* and *Golli-MBP*^*fl/*+^*; WT*) mice aged 52–53 weeks were assessed using an accelerating rotarod (#MK-610, Muromachi Kikai Co., Ltd., Tokyo, Japan). During training, mice were placed on a rotarod at a constant speed (4 rpm for 5 min). Mice that fell off during the training period were returned to the rotarod. Following training, three test sessions were performed using the accelerating speed levels of the apparatus (4–45 rpm) for 5 min. The latency to fall was recorded with a maximum latency of 5 min. The mice were given a 5-min intertrial rest between trials. The apparatus was wiped using a 70% ethanol solution and dried before each trial. The mean latency to fall off the rotarod was recorded and used for subsequent analyses.

### Footprint test

Gait disturbances in *Golli-MBP*^*fl/fl*^*; E3CreN, Golli-MBP*^*fl/*+^*; E3CreN,* and control (*Golli-MBP*^*fl/fl*^*; WT* and *Golli-MBP*^*fl/*+^*; WT*) mice aged 52–53 weeks were assessed using a footprint test. The mice were initially trained to walk along a 420-mm-long and 80-mm-wide runway (with 250-mm-high walls). To obtain footprints, the hind and forelimbs of each mouse were painted with different colors with red and black ink, respectively. Three test sessions were carried out, each followed by a training session. A white paper measuring 420 mm in length and 80 mm in width was placed on the runway floor for each run. Footprints were subsequently analyzed for four gait parameters measured in centimeters, as previously described (Carter et al. 1999): (1) stride length (average forward distance between each stride), (2) hindlimb base width, (3) forelimb base width (average distance between left and right hind and front footprints), and (4) front footprint/hindfootprint overlap (average distance left and right front footprint/hind footprint overlap). For each step parameter, three values were measured from each run, excluding footprints made at the beginning and end of the run when the animal was initiating and finishing movements, respectively. The mean of each set of three values was utilized in subsequent analyses.

### Statistics

GraphPad Prism version 8 was used for all statistical analyses. Data for image analysis are presented as the mean ± SD, and were analyzed using two-tailed unpaired *t* test with Welch’s correction. Data for qPCR, body weight, footprint test, and rotarod test were initially examined for outliers using Grubbs’ test, are presented as mean ± SD, and were analyzed by one-way ANOVA, followed by Tukey’s multiple-comparison test. *p < 0.05; **p < 0.01; and ***p < 0.001. Statistical significance was set at a *p* value < 0.05.

## Results

### Expression and localization of Classic-MBP and Golli-MBP in the mouse brain

**Fig. 1:**
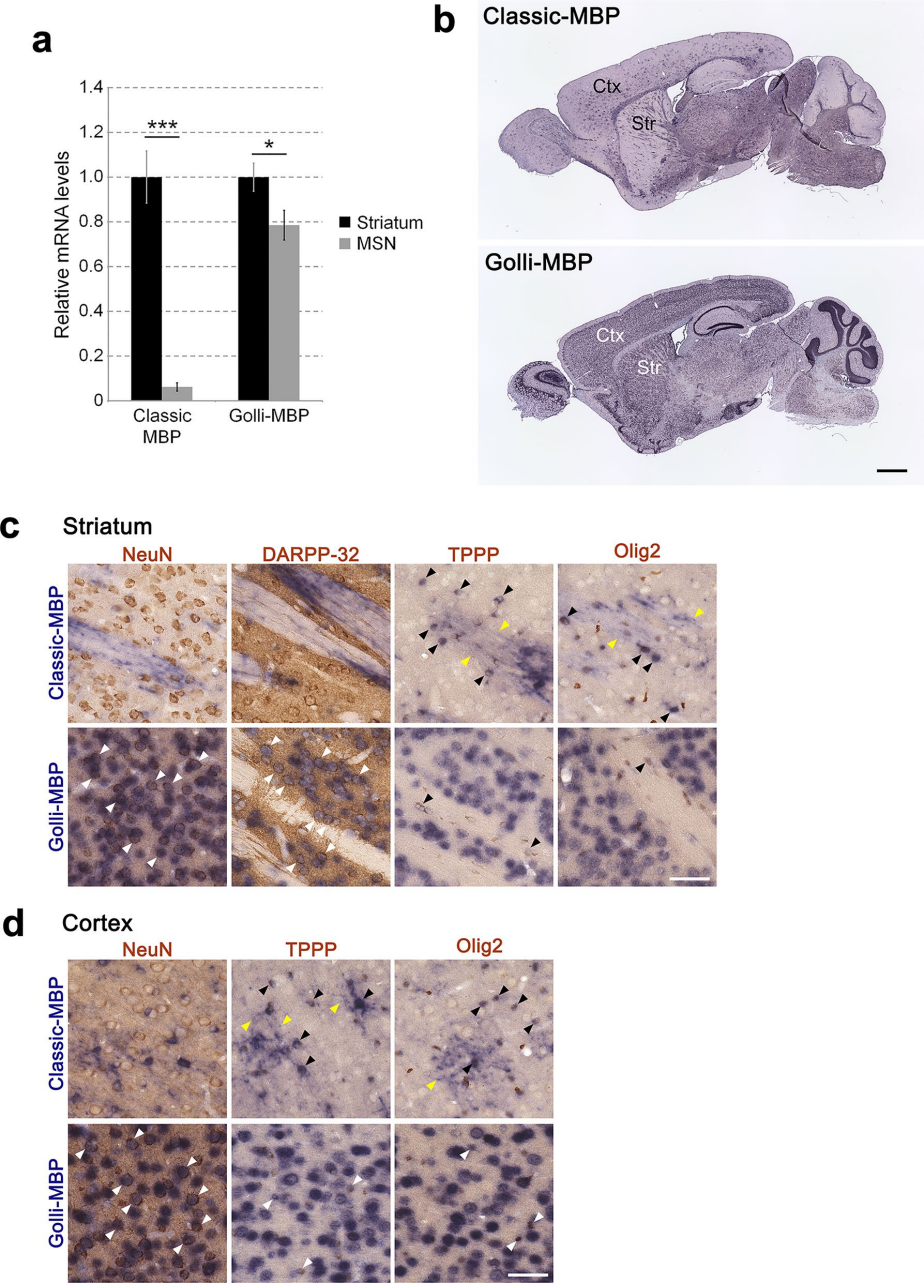
Expression and localization of Classic-MBP and Golli-MBP in the mouse brain. **a** Relative mRNA levels of Classic-MBP and Golli-MBP in whole striatal samples and FACS-purified MSNs. Data are presented as the mean ± SD. (*n* = 4 for whole striatal samples,* n* = 6 for MSNs). *Gapdh* was used to normalize the gene expression data. **b** mRNA distribution of Classic-MBP and Golli-MBP in sagittal mouse brain sections at 8 weeks of age. **c**, **d** Images of ISH-IH combined staining in the striatum (**c**) and cortex (**d**) of sagittal mouse brain sections at 8 weeks of age. Blue signals represent the mRNA distribution of Classic-MBP and Golli-MBP. Brown signals represent immunostaining signals by anti-NeuN, DARPP-32, TPPP, and Olig2 antibodies. Black and white arrows indicate double-labeled cells. Yellow arrows indicate diffuse and dotted signals of Classic-MBP mRNA distributed in the oligodendrocyte processes. Scale bar, 1 mm (**b**); 50 µm (**c**, **d**). Ctx, cortex; Str, striatum

One of the distinguishing characteristics of Golli-MBP compared with Classic-MBP is its expression in neurons. To verify whether Golli-MBP was expressed in neurons, we performed qPCR using whole striatal samples, which are a mixture of neurons and glial cells, and FACS-purified striatal MSNs, in which Golli-MBP was expressed (Landry et al. 1996). As expected, Classic-MBP expression was observed in whole striatal samples. However, expression was almost completely lost in FACS-purified MSNs (Fig. 1a). Classic-MBP expression is restricted to myelin-forming oligodendrocytes in the CNS. Thus, the expression was mostly deficient in MSNs. Conversely, Golli-MBP expression was observed in whole striatal samples and FACS-purified MSNs (Fig. 1a). This suggests that Golli-MBP is expressed in MSNs and other cell types in the striatum. We subsequently compared mRNA distribution between Classic-MBP and Golli-MBP in the mouse brain using ISH. The mouse brain images showed distinct mRNA distribution patterns between Golli-MBP and Classic-MBP (Fig. 1b). To confirm whether Classic-MBP or Golli-MBP isoforms were expressed in neuronal populations, we performed ISH-IH combined staining using cell type-specific markers (Fig. 1c, d). Classic-MBP mRNA is localized not only in the cell body but also in the processes of oligodendrocytes (Muller et al. 2013). To correctly assess the colocalization of Classic-MBP and cell-type specific markers, we only focused on mRNA signals in the cell body. Classic-MBP were not colocalized with immunohistochemical signals of anti-NeuN, which is a common neuronal marker (Gusel'nikova and Korzhevskiy 2015), and anti-DARPP-32, which is a widely used marker of striatal MSNs (Campbell et al. 1995), in the striatum and cortex (Fig. 1c, d). In contrast, the mRNA signals of Classic-MBP clearly colocalized with the immunohistochemical signals of anti-tubulin polymerization-promoting protein (TPPP), which is a marker of differentiated oligodendrocytes, and partially colocalized with the immunohistochemical signals of anti-Olig2, which is a marker of immature oligodendrocytes, in the striatum and cortex (Fig. 1c, d). These results suggest that Classic-MBP mRNA is localized in oligodendrocytes, which is consistent with a previous report (Holz and Schwab 1997). Golli-MBP mRNA signals were mainly colocalized with anti-NeuN and anti-DARPP-32 signals and partly colocalized with anti-TPPP and anti-Olig2 signals (Fig. 1c, d). A similar result was observed for ViewRNA ISH, which is a highly sensitive and specific branched fluorescence ISH (FISH) technology, using a Golli-MBP-specific probe set (Fig. S4). Golli-MBP mRNA foci were observed not only in pyramidal neurons of the hippocampal CA1 region, granule cells of the dentate gyrus, striatal neurons, layer 3 and layer 5 neurons of the cortex, and cerebellar granule neurons, but also in glial cells in the corpus callosum (Fig. S4). These results suggest that Golli-MBP is mainly expressed in neurons and partially expressed in oligodendrocytes.

### Generation of Golli-MBP-floxed mouse

**Fig. 2:**
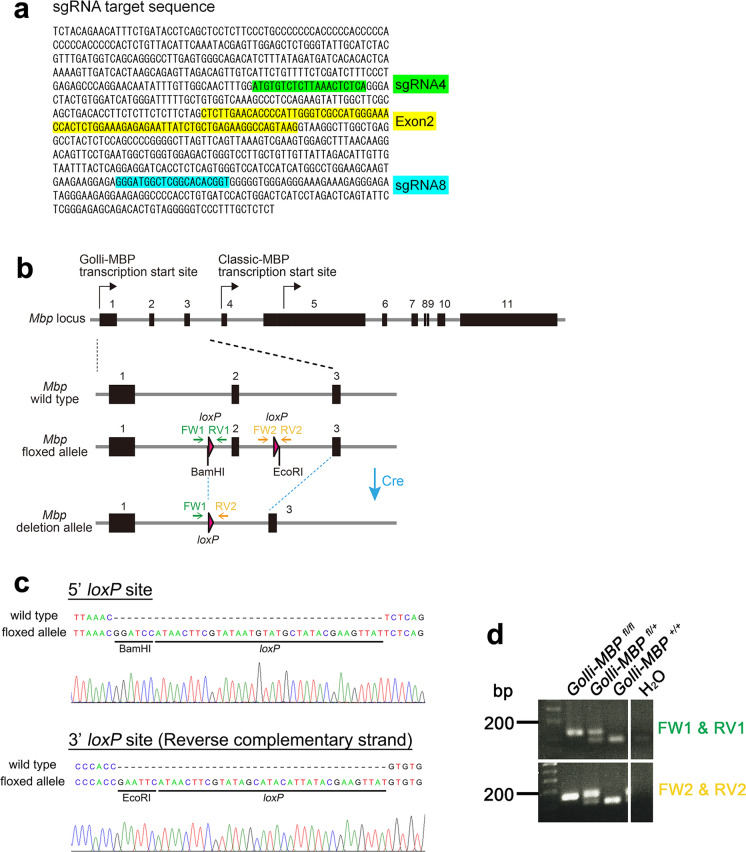
Generation of Golli-MBP-floxed mouse. **a** Genomic sequence of sgRNA targeting site in *Mbp*. Exon2 of the *Mbp* (yellow), left sgRNA targeting site (sgRNA4, green), and right sgRNA targeting site (sgRNA8, blue) are represented. **b** Diagram of the targeting strategy to ablate exon2 of the *Mbp*. The floxed allele of *Mbp* contains loxP sites (pink triangles) and artificially inserted restriction enzyme sites (*BamH*I, *EcoR*I). *Mbp* exon2 was removed by crossbreeding with Cre transgenic mouse. The regions of genotyping primers are shown as green allows (Golli-flox-FW1, Golli-flox-RV1) and yellow allows (Golli-flox-FW2, Golli-flox-RV2). **c** Sequence of 5’ and 3’ loxP site of line #40 Golli-MBP-floxed mouse. The sequences were verified by sequencing. **d** Genotyping PCR of Golli-MBP-floxed mouse using two primer sets that amplify two loxP sites individually. The upper band represents the floxed allele, and the lower band represents the wild-type allele

To investigate the cell-type–specific function of Golli-MBP, we generated a Golli-MBP-floxed mouse using CRISPR/Cas9 genome editing technology. sgRNAs were designed for exon 2 of the *Mbp* gene (Fig. 2a), because exon 2 deletion has been confirmed to result in selective ablation of Golli-MBP transcripts and proteins, but not Classic-MBPs (Jacobs et al. 2005). Highly efficient sgRNAs (sgRNAs 4 and 8) were selected using a modified method from a previous report (Mashiko et al. 2013) (Fig. 2a and Fig. S1; see Materials and Methods for details). The CRISPR/Cas9/sgRNA expressing plasmids and LoxP – MBP exon2 – LoxP containing plasmid were co-transfected into mouse ES cell lines derived from C57BL/6N (EGR-101). PCR genotyping and sequencing identified five ES cell lines (# 8, # 31, # 38, # 40, # 92) carrying a correctly targeted insert that displayed as “*Mbp* floxed allele” in Fig. 2b. After karyotype analysis, three clones, # 8, # 38, and #to 40, were selected for microinjection into 8-cell stage embryos of ICR mice. Targeted insertion in the F0 founder mice was verified by PCR genotyping and sequencing of the F1 offspring (Fig. S3 and Fig. 2c). Finally, we confirmed germline transmission of the targeted insert in line #40 Golli-MBP-floxed mice. PCR genotyping of Golli-MBP-floxed mice was performed by two specific primer sets: Golli-flox-FW1 and -RV1, Golli-flox-FW2 and -RV2 detect left and right loxP insertion individually (Fig. 2c, d).

### Generation of CGN-specific Golli-MBP gene knockout mouse

**Fig. 3:**
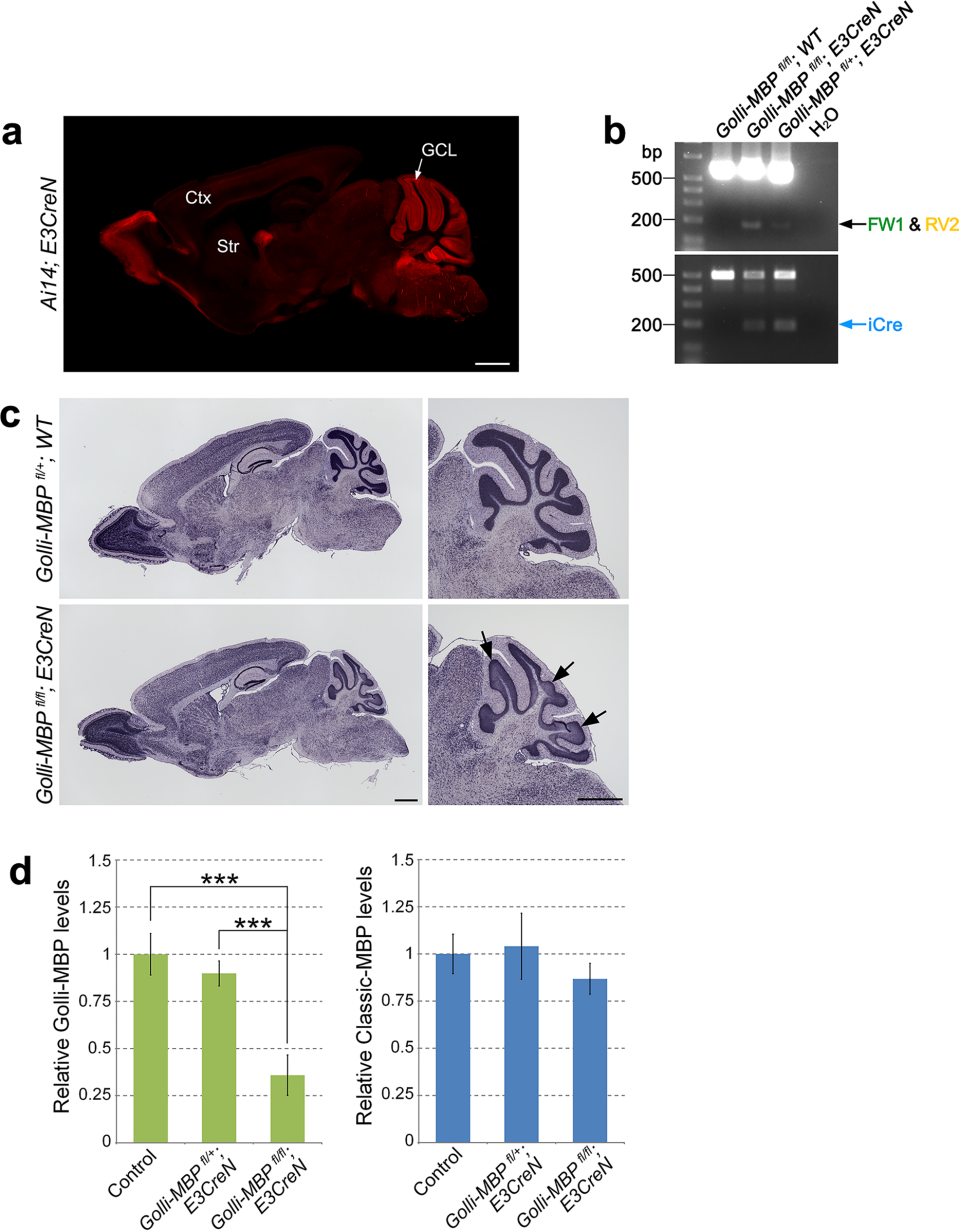
Characterization of *Golli-MBP*^*fl/fl*^*; E3CreN* mouse. **a** Distribution of iCre in the brain of *E3CreN* was determined by tdTomato expression of *Ai14; E3CreN* mouse at 8 weeks of age. **b** Genotyping PCR of *Golli-MBP*^*fl/fl*^*; E3CreN* mouse using two primer sets that amplify deletion allele (top) and iCre (bottom). The top panel shows the wild-type (upper band) and deleted exon2 (lower band) alleles. The bottom panel shows the wild-type allele (upper band) and iCre (lower band). **c** Images of ISH using Golli-MBP specific probe in the sagittal section of *Golli-MBP*^*fl/fl*^*; E3CreN* and control (*Golli-MBP*^*fl/*+^*; WT*) mice. The right panels show higher magnification figures of the cerebellum in the left panels. Arrows reveal decreased mRNA levels of Golli-MBP in CGNs of 8-week-old *Golli-MBP*^*fl/fl*^*; E3CreN* compared with control mouse. **d** Relative mRNA levels of Golli-MBP and Classic-MBP in the cerebellum of *Golli-MBP*^*fl/fl*^*; E3CreN*, *Golli-MBP*^*fl/*+^*; E3CreN,* and control (*Golli-MBP*^*fl/fl*^*; WT* and *Golli-MBP*^*fl/*+^*; WT*) mice. Data are presented as the mean ± S.D. (*error bars*) (n = 4 from each genotype). *Gapdh* was used to normalize the gene expression data. ***p < 0.001, one-way ANOVA followed by Tukey’s multiple-comparison test. Scale bar, 1 mm (**a**, **c** (left and right)). Ctx, cortex; Str, striatum; GCL, granule cell layer

To investigate the role of Golli-MBP in neurons, we established a conditional knockout mouse in which Golli-MBP expression was selectively ablated in CGNs, where Golli-MBP is strongly expressed during early postnatal development and adulthood (Landry et al. 1996). To ablate Golli-MBP in the CGNs, we used *E3CreN* mice expressing Cre in the CGNs (Miyazaki et al. 2012). To visualize Cre expression, *E3CreN* was crossed with Ai14, a Cre reporter mouse (Madisen et al. 2010). tdTomato expression in *Ai14; E3CreN* mice showed that Cre was abundantly expressed in the CGNs of the granule cell layer (GCL) of *E3CreN* mice (Fig. 3a). To assess Cre recombinase activity, *E3CreN* mice were crossed with *Scn2a*-floxed mice (*Scn2a*^*fl/fl*^*; E3CreN*) (Ogiwara et al. 2018) because the Nav1.2 voltage-gated sodium channel encoded by *Scn2a* gene was strongly expressed in parallel fibers, which are the axons of CGNs (Martínez-Hernández et al. 2013). Immunohistochemical analysis showed that Nav1.2 was abundantly expressed in the parallel fibers of the control mice (*Scn2a*^*fl/fl*^*; WT*) but was lost in *Scn2a*^*fl/fl*^*; E3CreN* (Fig. S5)*.* This result suggests that Cre-mediated homologous recombination and DNA deletion occurred with a high efficiency of Cre recombinase in mice. Homologous recombination at the *Mbp* deletion allele of *Golli-MBP*^*fl/fl*^*; E3CreN* was confirmed by PCR using the Golli-flox-FW1 and Golli-flox-RV2 primers (Fig. 3b and Table S4). In situ hybridization (ISH) using a Golli-MBP-specific probe showed that the expression levels of Golli-MBP mRNA decreased in the CGNs of *Golli-MBP*^*fl/fl*^*; E3CreN* mice (Fig. 3c). The qPCR findings revealed that Golli-MBP mRNA levels were significantly decreased in the cerebellum of *Golli-MBP*^*fl/fl*^*; E3CreN* mice (Fig. 3d). Conversely, the mRNA levels of Classic-MBP were not altered in the cerebellum of *Golli-MBP*^*fl/fl*^*; E3CreN* mice compared with control and heterozygous mice (Fig. 3d). We could not confirm the loss of Golli-MBP because we were unable to generate antibodies against Golli-MBP. However, our results strongly suggested that Golli-MBP proteins were absent in the CGNs of *Golli-MBP*^*fl/fl*^*; E3CreN* mice.

#### Immunohistochemical analysis of ***Golli-MBP***^***fl/fl***^;*** E3CreN*** mouse

**Fig. 4:**
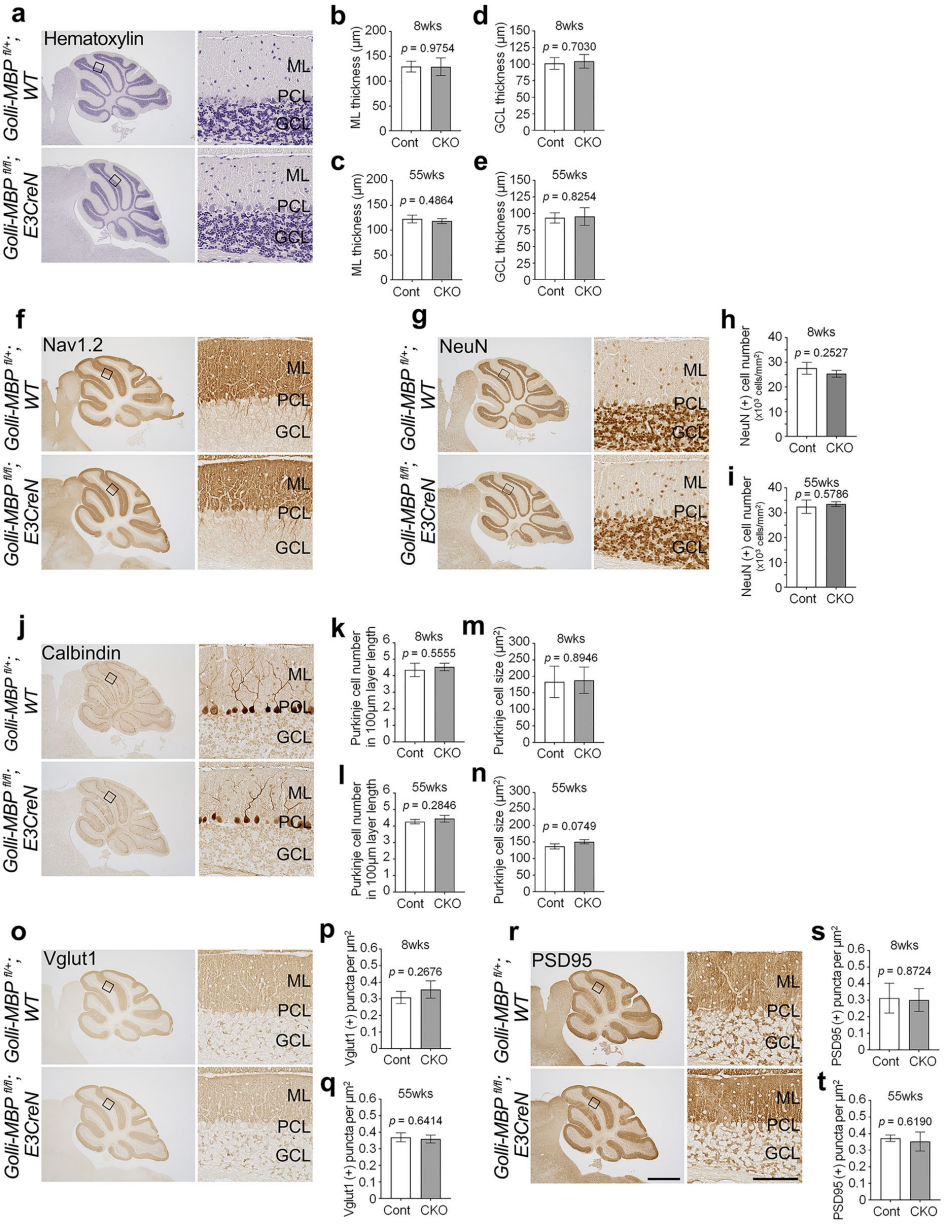
Quantitative histological analysis of the cerebellum in 8- and 55-week-old *Golli-MBP*^*fl/fl*^*; E3CreN* mouse. **a**, **f**, **g**, **j**, **o**, **r** Sagittal sections of 8-week-old *Golli-MBP*^*fl/fl*^*; E3CreN* and control (*Golli-MBP*^*fl/*+^*; WT*) mice stained with hematoxylin (**a**), anti-Nav1.2 (**f**), anti-NeuN (**g**), anti-calbindin D28K (**j**), anti-Vglut1 (**o**), and anti-PSD95 (**r**). Panels on the right show higher magnifications of the boxed areas in the left panels. Continuous sections of the same individual mice are shown. (**b-e**) Quantification of ML (**b, c**) and GCL (**d, e**) thickness in the 8-week-old (8wks) (**b, d)** and 55-week-old (55wks) (**c, e**) *Golli-MBP*^*fl/fl*^*; E3CreN* (CKO) and control (Cont) (8wks: *Golli-MBP*^*fl/*+^*; WT*, 55wks: *Golli-MBP*^*fl/fl*^*; WT*) mice. (**h, i**) Quantification of the number of NeuN-positive cells in GCL in 8wks (**h**) and 55wks (**i**) old in CKO and control mice. (**k-n**) Quantification of the number of Purkinje cells between 100µm PCL length (**k, l**) and cell body size (**m, n**) of Purkinje cells in 8wks (**k, m**) and 55wks (**l, n**) old CKO and control mice. (**p, q**) Quantification of the number of Vglut1-positive puncta in 8wks (**p**) and 55wks (**q**) old CKO and control mice. (**s, t**) Quantification of the number of PSD95-positive puncta in 8wks (**s**) and 55wks (**t**) old of CKO and control mice. Data are presented as the mean ± S.D. (error bars) (n = 3 for each genotype). Data were analyzed by two-tailed unpaired *t* test with Welch’s correction. Scale bars, 1 mm (left panels) and 100 μm (right panels). ML, molecular layer; PCL, Purkinje cell layer; GCL, granule cell layer

To assess the impact of cerebellar Golli-MBP deletion on CGNs, we initially examined the morphological changes in the cerebellum using hematoxylin staining (Fig. 4a). Overall, there were no significant differences in the thickness of the ML or GCL between *Golli-MBP*^*fl/fl*^*; E3CreN* and control mice (ML, 8-week-old: *p* = 0.9754; 55-week-old: *p* = 0.4864, unpaired two-tailed *t* test) (GCL, 8-week-old: *p* = 0.7030; 55-week-old: *p* = 0.8254, unpaired two-tailed *t* test) (Fig. 4b–e). To determine the morphological changes in the parallel fibers, we further performed immunostaining using an anti-Nav1.2 antibody, which binds to parallel fibers (Martínez-Hernández et al. 2013). Nav1.2 distribution in the parallel fibers of *Golli-MBP*^*fl/fl*^*; E3CreN* mice was similar to that observed in the parallel fibers of control mice (Fig. 4f), indicating that the parallel fibers in *Golli-MBP*^*fl/fl*^*; E3CreN* mice were intact. To further examine the alterations in the cerebellar neuronal population, we performed immunostaining using an anti-NeuN antibody (Fig. 4g). There was no significant difference in the number of stained CGNs between *Golli-MBP*^*fl/fl*^*; E3CreN* and control mice (8-week-old: *p* = 0.2527; 55-week-old: *p* = 0.5786, unpaired two-tailed *t* test) (Fig. 4h, i). We further examined the degeneration of Purkinje cells by immunostaining with an anti-calbindin D28K antibody, a specific marker of Purkinje cells (Afshar et al. 2017) (Fig. 4j). Quantification of the number and cell body size of Purkinje cells did not show any significant differences between the control and *Golli-MBP*^*fl/fl*^*; E3CreN* mice (number, 8-week-old: *p* = 0.5555; 55-week-old: *p* = 0.2846, unpaired two-tailed *t* test) (cell body size, 8-week-old: *p* = 0.8946; 55-week-old: *p* = 0.0749, unpaired two-tailed *t* test) (Fig. 4k–n). These results suggested that CGNs and Purkinje cells were not degenerated by Golli-MBP deletion.

Parallel fibers form excitatory synapses with the dendrites of Purkinje cells, which are widely known to be an essential site for long-term depression and potentiation (Hoxha et al. 2016). Parallel-fiber Purkinje cell (PF-PC) synapses have further been proposed to play a crucial role in motor learning (Hoxha et al. 2016). To examine presynaptic deficits at the PF-PC synapse, we analyzed the expression level and distribution of the presynaptic marker Vglut1, a vesicular glutamate transporter selectively expressed in parallel fiber synaptic varicosities (Hioki et al. 2003) (Fig. 4o). The number of Vglut1-positive puncta within the ML was not significantly different between *Golli-MBP*^*fl/fl*^*; E3CreN* and control mice (8-week-old: *p* = 0.2676; 55-week-old: *p* = 0.6414, unpaired two-tailed *t* test) (Fig. 4p, q). A similar result was obtained after staining with an antibody against PSD95, scaffold protein enriched in the postsynaptic density (Okabe 2007) (Fig. 4r). Quantification of the number of PSD95-positive puncta within the ML did not reveal any significant differences between *Golli-MBP*^*fl/fl*^*; E3CreN* and control mice (8-week-old: *p* = 0.8724; 55-week-old: *p* = 0.6190, unpaired two-tailed *t* test) (Fig. 4s, t). These results indicate that the loss of Golli-MBP did not affect PF-PC synapse formation and maintenance.

Neurodegeneration elicits abnormal responses in glial cells, particularly in astrocytes and microglia, which are highly sensitive to brain perturbations (Gleichman and Carmichael 2020). Therefore, we hypothesized that if the Golli-MBP deficit causes cerebellar perturbations, the number of “reactive” glial cells may increase. Immunohistochemical analysis for anti-GFAP, a classical marker of mature astrocytes, showed similar distributions of these cells in the cerebellum of *Golli-MBP*^*fl/fl*^*; E3CreN* and control mice (Fig. S6a). Immunohistochemical analysis using anti-IbaI, a microglial marker, further showed that a small number of IbaI-positive microglia existed in the ML and GCL in the cerebellum of *Golli-MBP*^*fl/fl*^*; E3CreN* and control mice (Fig. S6b). Quantification of the number of IbaI-positive microglia within the ML or GCL did not reveal any significant differences between the two groups of mice (ML, 8-week-old: *p* = 0.9348; 55-week-old: *p* = 0.8469, unpaired two-tailed *t* test) (GCL, 8-week-old: *p* = 0.9243; 55-week-old: *p* = 0.3779, unpaired two-tailed *t* test) (Fig. S6c, d). Furthermore, we examined the alterations in oligodendrocytes in the cerebellum. Immunostaining with an anti-MBP antibody showed that the expression levels of MBP and distribution of MBP-positive mature oligodendrocytes did not change in the cerebellum of *Golli-MBP*^*fl/fl*^*; E3CreN* mice (Fig. S6e). These results indicate that loss of Golli-MBP in CGNs does not induce cerebellar perturbations.

#### Behavior analysis of ***Golli-MBP***^***fl/fl***^;*** E3CreN*** mouse

**Fig. 5:**
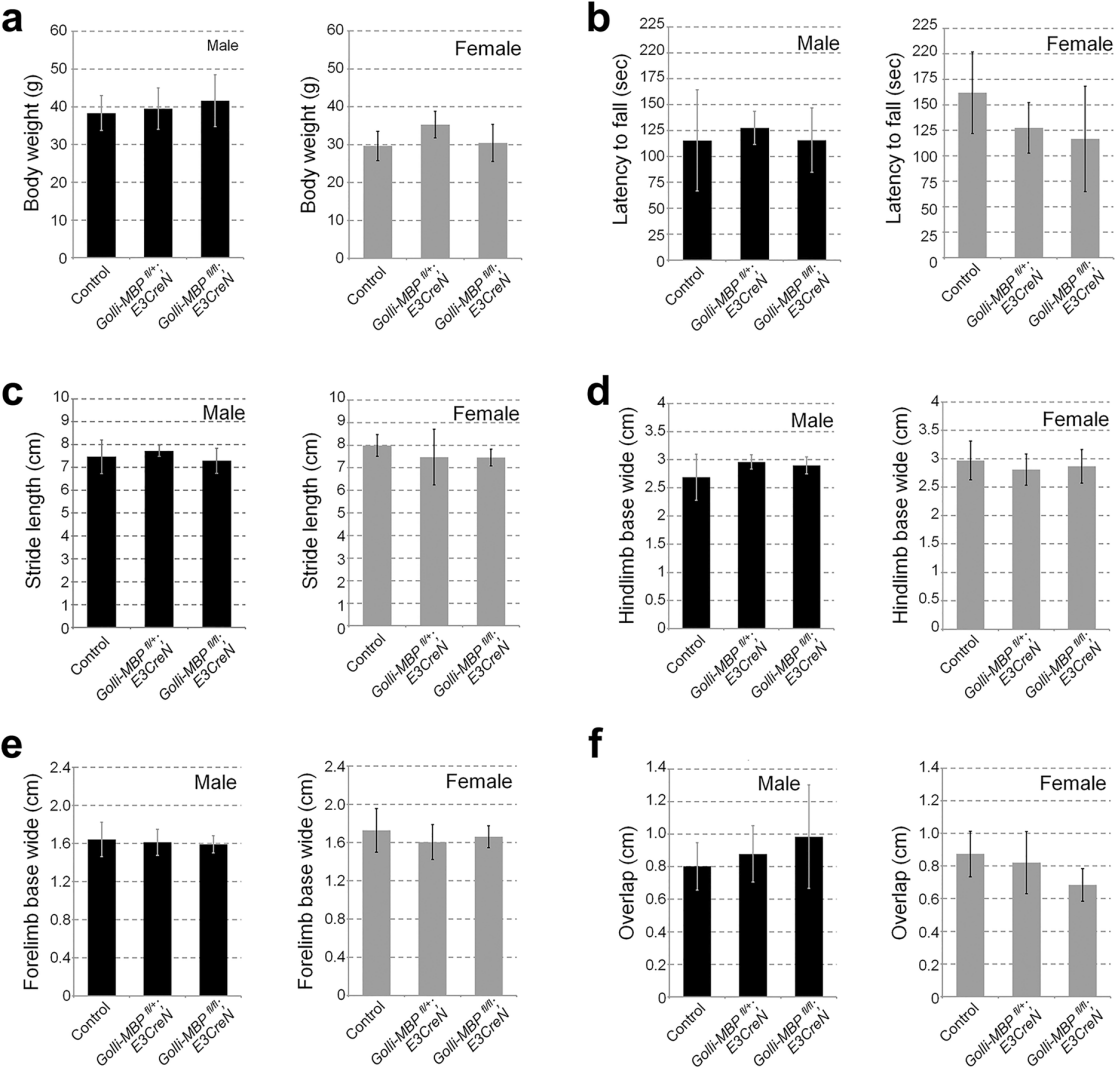
Body weight, rotarod test, and gait analysis of *Golli-MBP *^*fl/fl*^*; E3CreN* mice. **a** Body weight and **b** rotarod test of *Golli-MBP *^*fl/fl*^*; E3CreN, Golli-MBP *^*fl/*+^*; E3CreN* and control (*Golli-MBP *^*fl/fl*^*; WT* and *Golli-MBP *^*fl/*+^*; WT*) mice aged 52–53 weeks. **c**–**f** Gait disturbance was assessed by footprint test using four different parameters: **c** stride length, **d**, **e** hindlimb and forelimb base width, and **f** front footprint/ hind footprint overlap. The left bar graph (black) indicates male mice, and the right bar graph (gray) indicates female mice. Data are presented as the mean ± S.D. (error bars) (male: n = 7 for control, n = 4 for *Golli-MBP *^*fl/*+^*; E3CreN*, n = 5 for *Golli-MBP *^*fl/fl*^*; E3CreN*; female: n = 5 for control, n = 4 for *Golli-MBP *^*fl/*+^*; E3CreN*, n = 3 for *Golli-MBP *^*fl/fl*^*; E3CreN*). Data were analyzed by one-way ANOVA followed by Tukey’s multiple-comparison test

The cerebellum plays a crucial role in the regulation of motor movement and balance control. Damage to the cerebellum leads to loss of motor movement coordination, gait disturbance, tremors, and weak muscles (Manto et al. 2012; De Zeeuw and Ten Brinke 2015). Young afibers of the cerebellumdult *Golli-MBP*^*fl/fl*^*; E3CreN* mice were healthy and exhibited no apparent abnormalities (data not shown). Thus, we assessed the behavior of 12-month-old *Golli-MBP*^*fl/fl*^*; E3CreN* mice to determine whether Golli-MBP loss in CGNs affects cerebellar function. The older *Golli-MBP*^*fl/fl*^*; E3CreN* mice exhibited no significant difference in body weight compared with the heterozygous and control mice (Fig. 5a). We performed the accelerated rotarod and footprint tests, which are standard assays widely used to evaluate rodent motor performance and gait disturbances, respectively (Hamm et al. 1994; Deacon 2013; Sugimoto and Kawakami 2019; Pallier et al. 2009). The latency to fall off the rotarod was similar in *Golli-MBP*^*fl/fl*^*; E3CreN* and control mice (Fig. 5b). The results of the footprint test, such as stride length, hindlimb base width, forelimb base width, and overlap, in *Golli-MBP*^*fl/fl*^*; E3CreN* mice showed no differences compared with heterozygous and control mice (Fig. 5c-f). These results suggest that loss of Golli-MBP in CGNs does not affect cerebellar function.

## Discussion

Golli-MBP, encoded by the *Mbp* gene, is widely expressed in neurons and oligodendrocytes in the CNS, during postnatal development and in adulthood. This study revealed the detailed localization of Golli-MBP mRNA in neurons and oligodendrocytes. ISH-IH combined staining and ViewRNA ISH results showed that Golli-MBP mRNA was prominently expressed in the striatal MSNs, granule neurons of the dentate gyrus, and cerebellar granule neurons in the mouse brain (Fig. 1c, d, and Fig. S4). Notably, the axonal fibers of these neurons, including the projection fibers of MSNs, the mossy fibers of the hippocampus, and the parallel fibers of the cerebellum, primarily comprise unmyelinated axons (Westenbroek et al. 1989; Miyazaki et al 2014). Thus, Golli-MBP was found to be notably enriched in unmyelinated fibers of the CNS. This observation raises the possibility that Golli-MBP plays a role in the regulation of unmyelinated fibers in the CNS.

In this study, we established Golli-MBP-floxed mice to gain insights into the cell type-specific functions of Golli-MBP. Our focus shifted to CGNs, which exhibit higher Golli-MBP expression levels than other brain regions (Fig. 1b), ultimately leading us to generate CGN-specific Golli-MBP knockout mice (*Golli-MBP*^*fl/fl*^*; E3CreN*) (Fig. 3). These *Golli-MBP*^*fl/fl*^*; E3CreN* mice displayed a significant decrease in Golli-MBP expression without any effect on Classic-MBP expression (Fig. 3d). Although *Golli-MBP*^*fl/fl*^*; E3CreN* mice exhibited a considerable reduction in Golli-MBP expression, histological analysis revealed no obvious morphological changes in the cerebellum (Fig. 4a-e). Immunohistochemical analysis using specific markers for parallel fibers, Purkinje cells, excitatory synapses, and glial cells showed no discernible alterations in the cerebellar components of *Golli-MBP*^*fl/fl*^*; E3CreN* mice compared to control mice (Fig. 4f-t and Fig. S6). Behavioral analysis further revealed that the loss of Golli-MBP in CGNs did not affect cerebellar function (Fig. 5). Thus, our investigation revealed no apparent functional or pathological abnormalities in the CGNs and cerebellum of *Golli-MBP*^*fl/fl*^*; E3CreN* mice, although it seems probable that functional redundancy arises in the CGNs, rather than negating the role of Golli-MBP in the development and function of CGNs. Previous studies have further shown that conventional Golli-MBP knockout mice exhibit delayed Classic-MBP expression and restricted hypomyelination of the visual cortex (Jacobs et al. 2005). However, transgenic JOE mice overexpressing the Golli-MBP isoform J37 in oligodendrocytes show a widespread delay in oligodendrocyte maturation, with associated hypomyelination throughout the brain (Jacobs et al. 2009). Cell-specific knockout of oligodendrocytes in our mice is important to resolve this contradiction.

It should further be noted that Golli-MBP is also expressed by the immune system, particularly in T lymphocytes (Feng 2007; Xu et al. 2016). Previous studies have proposed that Golli-MBP negatively regulates T-lymphocyte proliferation and activation through mechanisms involving the modulation of calcium homeostasis (Feng et al. 2004, 2006). Thus, our Golli-MBP-floxed mice are a valuable tool for studying the role of Golli-MBP in the immune system. Further studies are necessary to investigate the effect of Golli-MBP loss in other cell types such as other neuronal cells, oligodendrocytes, or lymphocytes.

### Supplementary Information

Below is the link to the electronic supplementary material.

Supplementary file1 (DOCX 1598 kb)

Supplementary file2 (XLSX 21 kb)
