# Supplementary material for: Generation and characterization of cerebellar granule neurons specific knockout mice of Golli-MBP
Source: Transgenic Res. 2024 Apr 29;33(3):99–117. doi: 10.1007/s11248-024-00382-0 (PMC11176102; doi:10.1007/s11248-024-00382-0)
Supplement: Supplementary file 1 — Supplementary file1 (DOCX 1598 kb) [file 11248_2024_382_MOESM1_ESM.docx]

**Transgenic Research**

**SUPPORTING INFORMATION**

Generation and characterization of cerebellar granule neurons specific knockout mice of Golli-MBP

Haruko Miyazaki*, Saki Nishioka, Tomoyuki Yamanaka, Manabu Abe, Yukio Imamura, Tomohiro Miyasaka, Nobuto Kakuda, Toshitaka Oohashi, Tomomi Shimogori, Kazuhiro Yamakawa, Masahito Ikawa, Nobuyuki Nukina*

**CONTENTS**

**Supplementary Figure 1.** Selection of the highly efficient sgRNAs for CRISPR/Cas9 genome editing.

**Supplementary Figure 2.** Schematic of CRISPR/Cas9 targeting site and targeting plasmid in *Mbp* exon2.

**Supplementary Figure 3.** Locus of genotyping primers for ES cells and founder mice.

**Supplementary Figure 4.** Distribution of Golli-MBP mRNA in the mouse brain.

**Supplementary Figure 5.** Assessment efficiency of Cre-mediated recombination in the CGNs of *E3CreN* mouse.

**Supplementary Figure 6.** Histological analysis of glial cells in the *Golli-MBP ^fl/fl^; E3CreN* mouse cerebellum.

*Corresponding author:

Haruko Miyazaki

Department of Molecular Biology and Biochemistry, Okayama University Graduate School of Medicine, Dentistry and Pharmaceutical Sciences, 2-5-1 Shikata-cho, Kita-ku, Okayama 700-8558, Japan

e-mail: hmiyazak@okayama-u.ac.jp

Tel: +81-86-235-7128

Fax: +81-86-222-7768

Nobuyuki Nukina

Laboratory of Structural Neuropathology, Graduate School of Brain Science, Doshisha University, 1-3 Tatara Miyakodani, Kyotanabe-shi, Kyoto 610-0394, Japan

e-mail: nnukina@juntendo.ac.jp


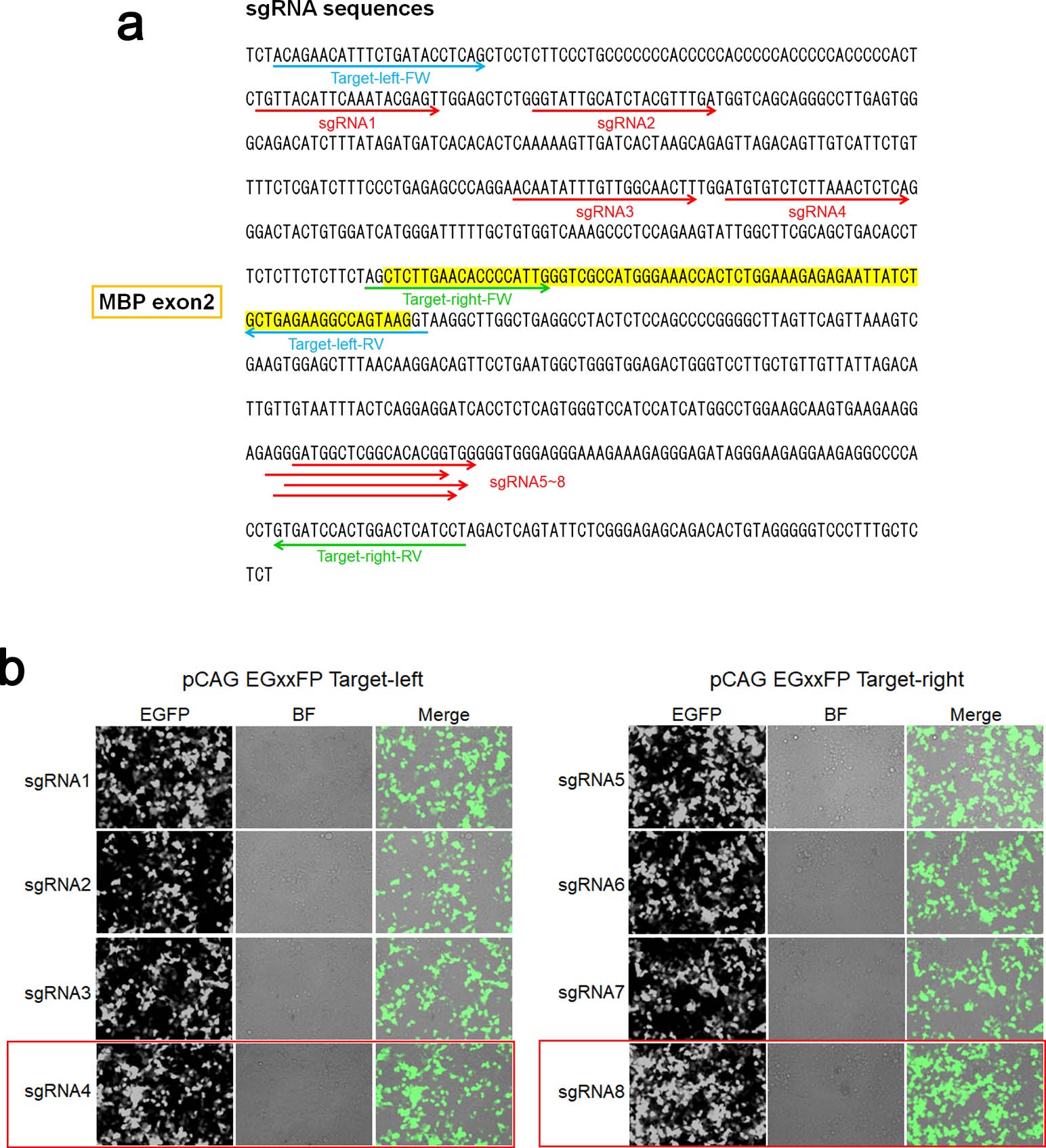


**Supplementary Figure 1. Selection of the highly efficient sgRNAs for CRISPR/Cas9 genome editing.** (**a**) Location and sequences of candidate sgRNAs (red) in the MBPs exon2 locus (yellow). These sgRNAs were amplified using specific primers (Table S1) and inserted into the pSpCas9 (BB)-2A-Puro (PX459) V2.0 vector. The left and right sgRNA target sites were amplified using Target-left-FW and -RV primers (blue) and Target-right-FW and -RV primers (green), respectively. The amplified target sites were inserted into the pCAG-EGxxFP vector. (**b**) Comparison of sgRNA efficiency at the target left site (sgRNA #1-4) and target right site (sgRNA #5-8). Plasmids of sgRNA and its target sites were co-transfected into HEK293T cells. sgRNA4 and sgRNA8 exhibited the highest GFP intensity among the sgRNAs. BF, Bright-field.


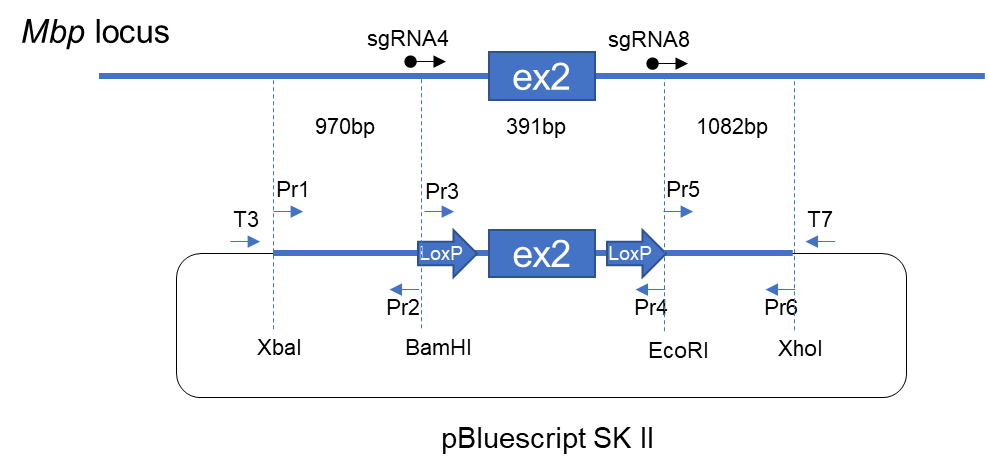


**Supplementary Figure 2. Schematic of CRISPR/Cas9 targeting site and targeting plasmid in *Mbp* exon2.** Selected sgRNA (4 and 8) sites in *Mbp* are indicated as black arrows. LoxP sites flanking *Mbp* exon2 (blue box) are shown in large blue arrows. Primers used to amplify DNA fragments comprising the targeting plasmid are shown as small blue arrows (Pr1-6). The PCR primer sets amplified the left, center, and right DNA fragments: Pr1 and Pr2 (left), Pr3 and Pr4 (center), Pr5 and Pr6 (right). T3 and T7 primers were used for sequencing. The sequence of these primers is provided in Table S2.


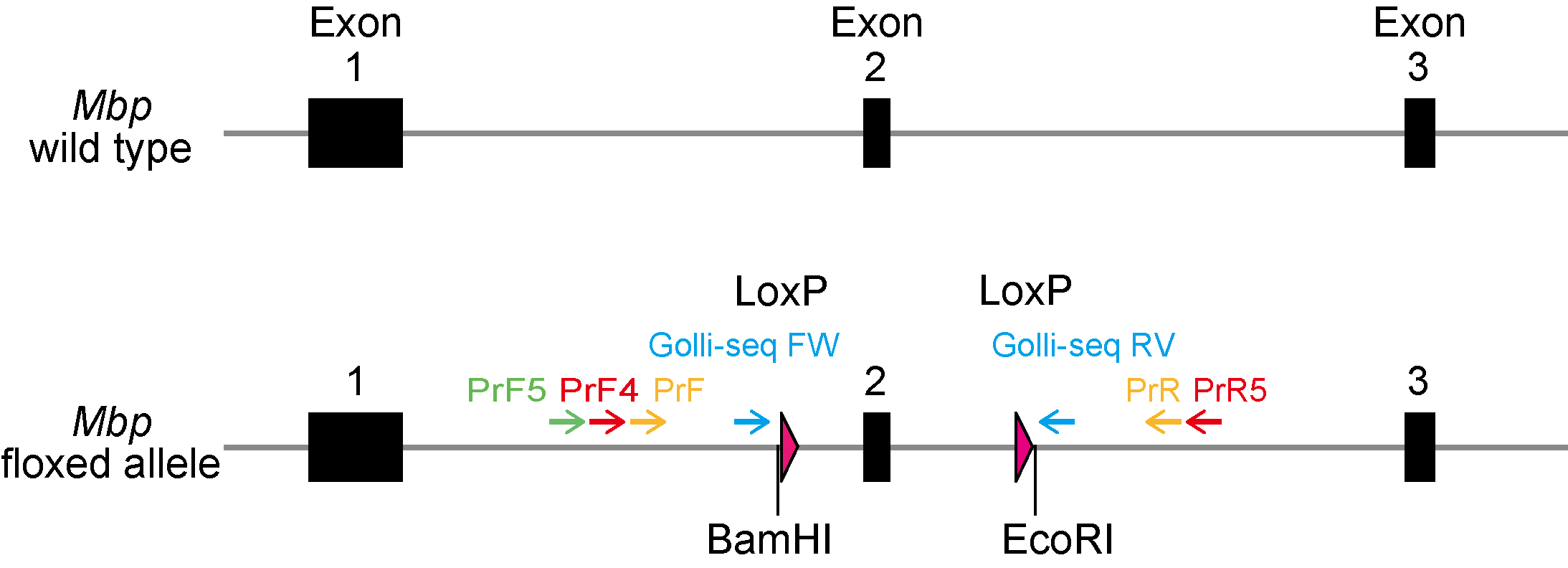


**Supplementary Figure 3. Locus of genotyping primers for ES cells and founder mice.** (Top) Diagram of the area around exon2 in wild-type *Mbp*. (Bottom) Diagram of the area around exon2 in the floxed *Mbp* allele. LoxP sites flanking *Mbp* exon2 are indicated as pink triangles. Genotyping of ES cells was performed by nested PCR. The first PCR was performed using PrF4 and PrR5 primer (red). The PCR products were used for the second PCR using PrF and PrR primers (yellow). The genotype of the offspring of founder mice was verified by PCR using primers Golli-PrF5 (green) and Golli-PrR (yellow). The sequence of the targeted site was confirmed by sequencing using Golli-seq FW and Golli-seq RV primers (blue). Primer sequences are listed in Table S3.


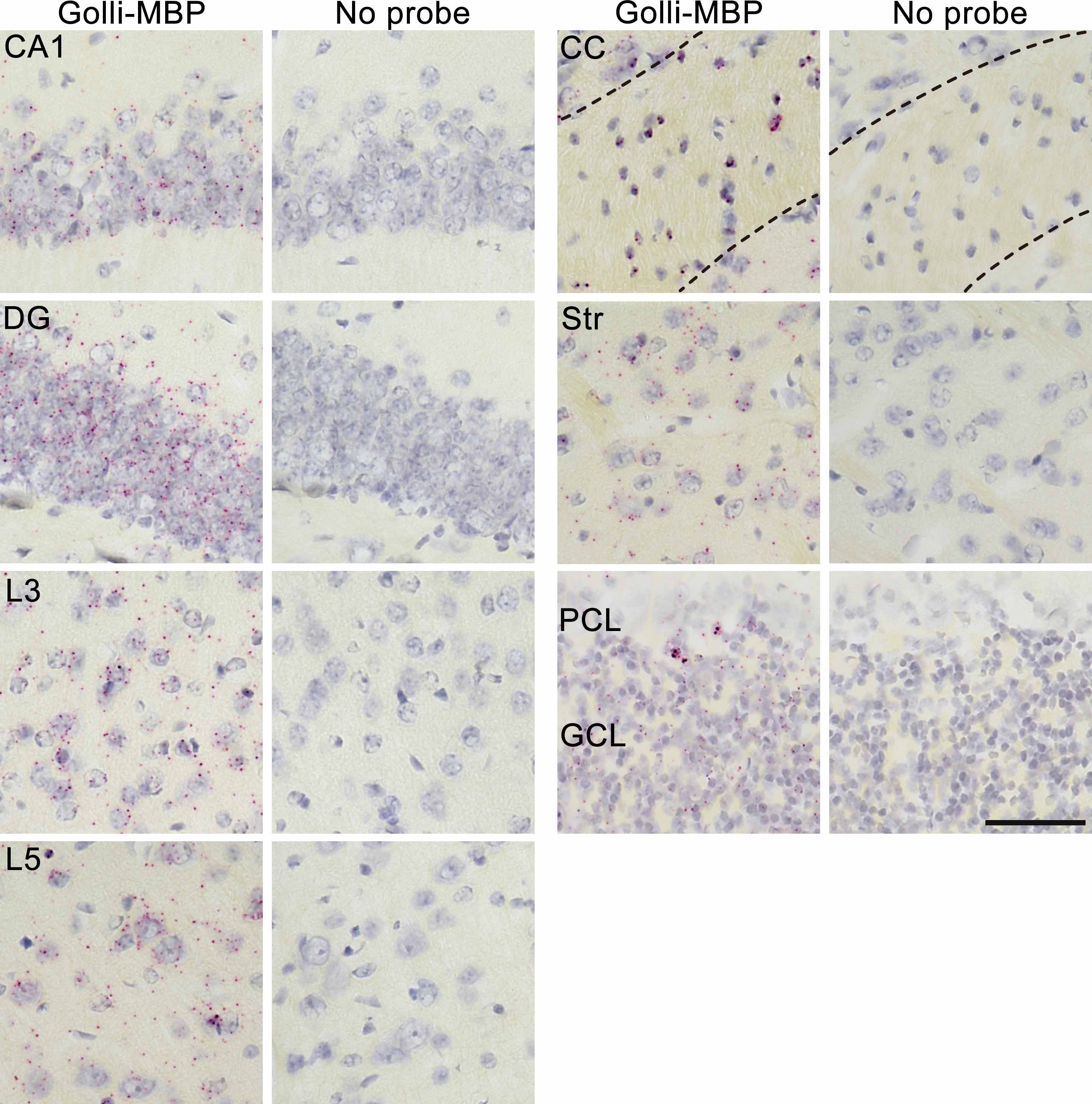


**Supplementary Figure 4. Distribution of Golli-MBP mRNA in the mouse brain.** ViewRNA signals of Golli-MBP mRNA (red) and Hematoxylin (blue) in sagittal brain sections of a C57BL/6J mouse aged 8 weeks. The left panels show ViewRNA signals of Golli-MBP, and the right panels show no probe control. Scale bar, 50µm. DG, dentate gyrus; L3, Cortex layer 3; L5, Cortex layer 5; CC, corpus callosum; Str, striatum; PCL, Purkinje cell layer; GCL, granule cell layer.


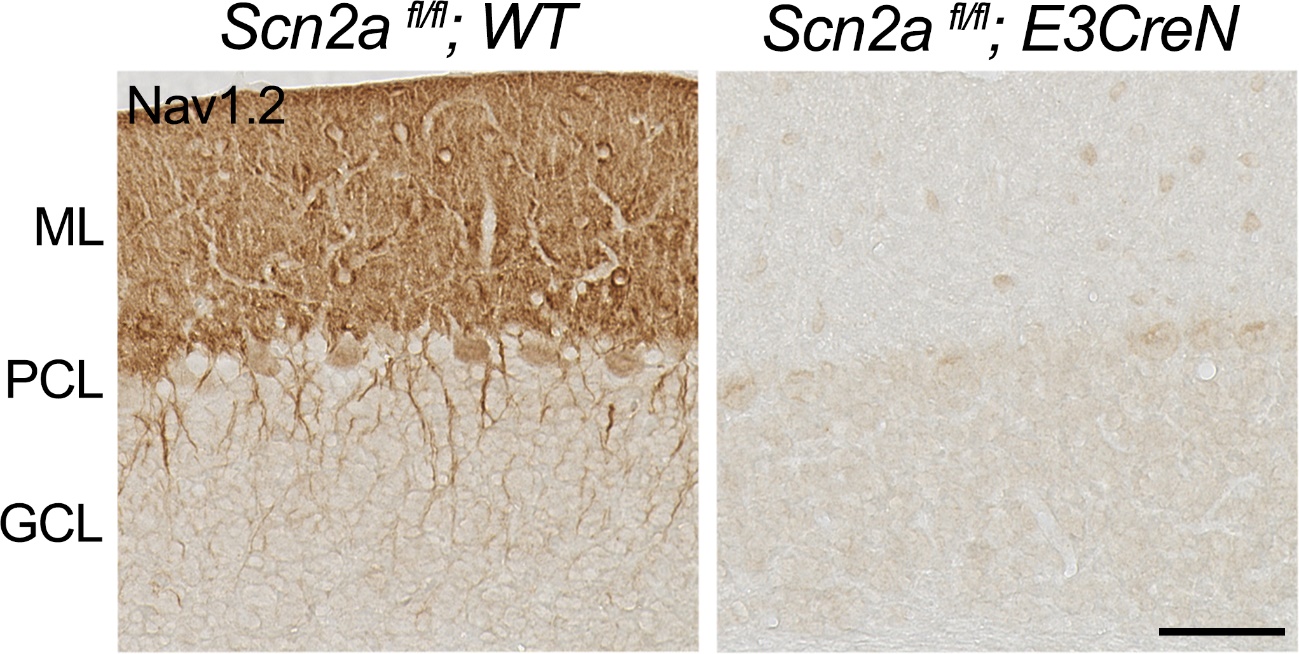


**Supplementary Figure 5.** **Assessment efficiency of Cre-mediated recombination in the CGNs of *E3CreN* mouse.** Immunohistochemical analysis of anti-Nav1.2 in the cerebellum of 20-week-old *Scn2 ^fl/fl^; E3CreN* and its control mouse (*Scn2a^fl/fl^; WT*). Nav1.2 encoded by *Scn2a* gene was strongly expressed in the parallel fiber of CGN in the control mouse (left panel), whereas Nav1.2 levels were lost in the parallel fiber of *Scn2a ^fl/fl^; E3CreN* mouse (right panel). Scale bar, 50µm. ML, molecular layer; PCL, Purkinje cell layer; GCL, granule cell layer.


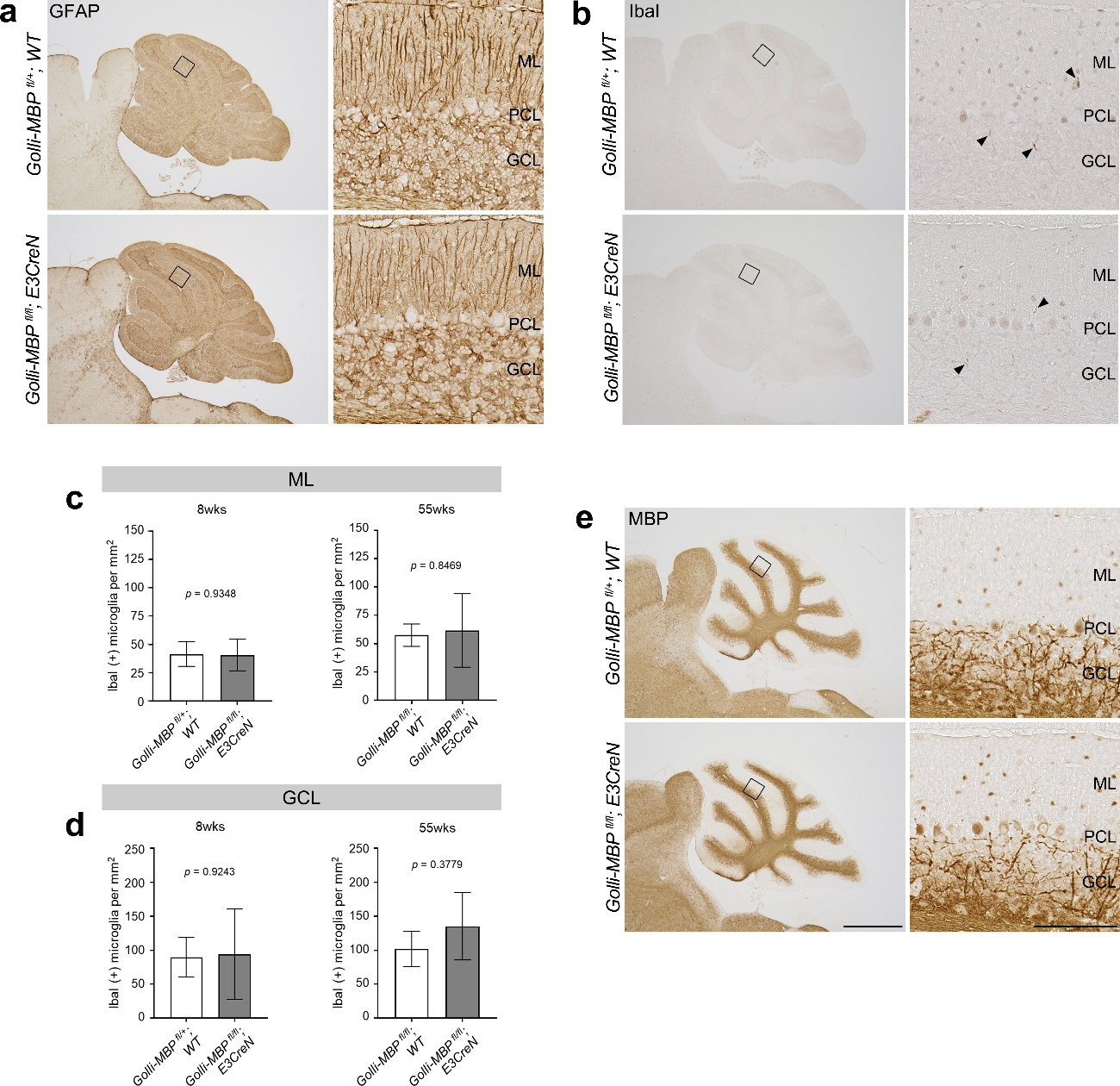


**Supplementary Figure 6. Histological analysis of glial cells in the *Golli-MBP ^fl/fl^; E3CreN* mouse cerebellum.** (**a, b, e**) Sagittal sections of 8-week-old *Golli-MBP ^fl/fl^; E3CreN* and control (*Golli-MBP ^fl/+^; WT*) mice were stained with anti-GFAP (**a**), anti-IbaI (**b**), and anti-MBP (**e**). Continuous sections of the same individual mouse were used. Pannels on the right show higher magnifications of the boxed areas in the left panels. Black arrowheads indicate the process and cell body of IbaI-positive microglia. (**c, d**) The number of IbaI-positive microglia in the ML (**c**) and GCL (**d**) of 8-week-old (8wks) and 55-week-old (55wks) *Golli-MBP ^fl/fl^; E3CreN* and control (*Golli-MBP ^fl/+^; WT* or *Golli-MBP ^fl/fl^; WT*) mice. Data are presented as the mean ± S.D. (error bars) (n = 3 for each genotype). Data were analyzed by two-tailed unpaired *t* test with Welch’s correction. Scale bars, 1mm (left panels) and 100µm (right panels). ML, molecular layer; PCL, Purkinje cell layer; GCL, granule cell layer.
